# Particle Imaging Velocimetry Gyroscope

**DOI:** 10.3390/s19214734

**Published:** 2019-10-31

**Authors:** Ahmed A. Youssef, Naser El-Sheimy

**Affiliations:** Department of Geomatics Engineering, Schulich School of Engineering, University of Calgary, Calgary, AB T2N 1N4, Canada; elsheimy@ucalgary.ca

**Keywords:** angular rate sensors, fluid-based inertial sensors, gyroscope, inertial measurement units, particle imaging velocimetry, particle tracking velocimetry

## Abstract

Inertial measurement units (IMUs) are typically classified as per the performance of the gyroscopes within each system. Consequently, it is critical for a system to have a low bias instability to have better performance. Nonetheless, there is no IMU available commercially that does not actually suffer from bias-instability, even for the navigation grade IMUs. This paper introduces the proposition of a novel fluid-based gyroscope, which is referred to hereafter as a particle imaging velocimetry gyroscope (PIVG). The main advantages of the PIVG include being nearly drift-free, a high signal-to-noise ratio (SNR) in comparison to commercially available high-end gyroscopes, and its low cost.

## 1. Introduction

Navigation is a process by which a moving platform determines its state of motion through what is termed as the navigation states. The navigation states for any given platform are the position, velocity, and attitude of such a platform. The navigation states should be able to characterize the motion of any given body in either a two-dimensional (2D) or three-dimensional (3D) space [1].

Navigation is performed through various methods; such methods are characterized mainly into two clusters: position fixing and dead reckoning. Position fixing is the process by which the navigation states are driven through the knowledge and use of a set of fixated well-defined positions. An example of position fixing navigation is the global navigation satellite system (GNSS). Whereas, dead reckoning is a process by which the navigation states are determined through a recursive process of relative positioning with respect to the initial states. An example of dead reckoning navigation is the inertial navigation system (INS) [1]. 

Inertial navigation is performed by processing the data of inertial measurement units (IMUs). IMUs are an assembly of sensors that are mounted in a geometric form that guarantees capturing the motion of any given platform. Such sensors measure what is referred to as inertial measurements and they include accelerometers, which measure specific forces (i.e., linear accelerations), and gyroscopes, which measure angular rates. The inertial measurements undergo a mathematical process, known as INS mechanization, that mainly includes integrations and geometric transformations to determine the navigation states of the moving platform with respect to a pre-defined reference frame. A typical IMU constitutes a triad of accelerometers and a triad of gyroscopes, placed along three mutually orthogonal axes.

Inertial sensors endure systematic errors which can be calibrated in the laboratory and eliminated from the raw inertial measurements. Such systematic errors include biases, scale factor, scale factor non-linearity, and cross-coupling. The bias is a constant error, whereby the inertial sensor reads out a measurement that is at a constant offset from the actual value. The scale factor is an error that is demonstrated by the existence of a gradient for the input–output relation of the inertial sensor which is not unity. However, if such a gradient is non-linear, then it is said that the inertial sensor suffers from scale factor non-linearity. Cross-coupling error indicates that a given inertial sensor (i.e., accelerometer or gyroscope) measures residual inertial measurements from an axis that is orthogonal to its sensitive axes. Typically, cross-coupling occurs due to the non-orthogonality of the sensitive axis of the inertial sensors [2].

Furthermore, inertial sensors suffer from random errors that appear as noises within the inertial measurements from electrical and mechanical sources. The noises are assumed to be white for frequencies below 1 Hz. The order of magnitude of such noises depends on the technology behind the manufacturing of the inertial sensor.

Nonetheless, the performance of any given IMU is defined by the order of magnitude of the systematic and random errors. Whereby, IMUs are classified according their performance and accuracy into various categories.

Consequently, IMUs are classified into strategic grade, navigation grade, tactical grade, automotive grade, and low-cost consumer grade IMUs. Strategic grade, also referred to as marine grade, is the highest quality IMUs. Strategic grade IMUs can be used in military ships, submarines, some intercontinental ballistic missiles, and some spacecraft. A strategic grade IMU might cost more than $1 million. Navigation grade IMUs are used in military aircrafts and commercial aviation. Navigation grade IMUs cost around $100,000. Tactical grade IMUs can only be used for stand-alone inertial navigation for a few minutes. However, an accurate navigation solution can be obtained by integration of the tactical grade IMU with an aiding positioning system. Typically, tactical grade IMUs are used in guided weapons and unmanned air vehicles (UAVs). Tactical grade IMUs cost around $2000 to $50,000. The automotive and consumer grade IMUs are the lowest grade of inertial sensors. They are typically used in pedometers, antilock braking, active suspension, and airbags. Consumer grade accelerometers costs start around $1, and the gyroscopes costs start around $10 [2].

Nevertheless, IMU grades are analogous to the technologies adopted to manufacture their inertial sensors (i.e., accelerometers and gyroscopes). Typical commercialized gyroscopes are classified into optical gyroscopes or vibratory gyroscopes. Optical gyroscopes utilize the phase of light waves to determine angular rates. The optical gyroscopes are realized by either ring laser gyroscope (RLG) technology or interferometric fiber-optic gyro (IFOG) technology [2]. Vibratory gyroscopes are low-cost, and often depend on Micro-electro-mechanical system (MEMS) technology, in which quartz-based gyros provide better performance than silicon-based gyros. There are other types of gyroscopes, which include nuclear magnetic resonance gyroscope technology, and cold-atom interferometry. Both technologies provide relatively higher performance at a higher cost [2,3].

A main characteristic for the inertial navigation system is the bias instability, regardless of the grade of said INS. In other words, the navigation solution that is acquired from inertial navigation systems tends to drift from the actual occupied trajectory due to variations in the bias offset value, which is referred to as bias instability. However, such bias instability is less the higher the grade of the IMU, but it is inevitable. The bias instability tends to be a stochastic error that cannot be evaluated directly using a mathematical model, which makes handling its effects a sophisticated aspect of research in inertial navigation.

## 2. Related Work

There are a lot of reported sensors that operate with utilizing fluid as the inertial proof mass of said sensors. Other sensors use fluids as a part of the inertial sensing mechanism. This section provides a brief overview on reported inertial angular motion sensors that use fluids within their process of measuring angular rates.

A type of inertial sensors that utilizes fluids as a part of its inertial sensing mechanism is the rate-integrating gyroscope (RIG). The rate-integrating gyroscope originally occurred in the 1960s. The rate-integrating gyroscope relies on the conservation of momentum and the trade-off between spatial rigidity and precession of a high-rate spinning rotor in 3-D space. One embodiment of the rate-integrating gyroscopes includes a high-rate spinning rotating body, with sufficient moment of inertia about its spin axis, should maintain a fixed spin axis in 3D space, unless acted upon by an external torque, which would lead the rotor to precess, such that the spin axis would follow the direction of the applied torque axis [4]. Many United States (US) patents were introduced in this period, developing various versions of rate-integrating gyroscopes (see [5,6,7,8,9,10]). 

Another type of fluid-based angular rate sensor is the magnetohydrodynamic (MHD) angular rate sensor. MHD angular rate sensors deploy the magnetohydrodynamic properties of fluid to measure the angular rate [4]. The magnetohydrodynamic effect implies that magnetic fields can induce electric currents in a moving conductive fluid (such as mercury). The induced current can polarize the flow, which in turn affects the applied magnetic field itself. The magnetohydrodynamic effect can be mathematically modeled using the Navier–Stokes equations for momentum conservation for incompressible fluids, and Maxwell’s equations for electromagnetism. The variations in the magnetic field are detected as an indication of the external angular motion imparted to these types of sensors.

Dual axis rate transducer (DART) is another form of a sensor that uses fluid as a part of its inertial sensing mechanism. This type of angular rate sensors first occurred in the 1960s in the United States. The sensor operation concept utilizes the law of conservation of momentum for a fluid body subjected to external inertial forces. The DART deploys a sphere of heavy liquid, such as mercury, as the sensing inertial element. The fluid sphere is contained within a spherical cavity. The spherical cavity is rotated via a driver motor at high speed such that the fluid sphere maintains a high angular momentum. Deflectable paddles are fixed at the periphery of the spherical cavity and passing through the fluid sphere. The paddles are attached to piezoelectric crystals. The paddles are used as a motion transduction mechanism. From the nomenclature of the sensor, it is noted that the device is sensitive to rotation about the two orthogonal axes, which are normal to the spin axis of the spherical cavity [4].

Jet flow gyroscopes are a type of fluid-based inertial angular rate sensors. The development of these type of sensors occurred in the 1960s. The scientific concept behind fluid flow rate sensors is utilizing the fluid to be the inertial sensing element, instead of using the fluid to drive or support a mechanical element. The sensors deploy the concept of temperature or pressure variations on a fluid control volume under the impact of external motion imparted to the control volume. These temperature and pressure variations are modeled by what is known as the Navier–Stokes equations, which describe the law of conservation of momentum for a fluid mass. Nonetheless, mathematical evaluation of the Navier–Stokes equations is not a straightforward problem. Hence, the detection of the external angular rate imparted to these kinds of sensors is dependent on the proportionality between the external motion and the temperature or pressure variations that occur within the sensor internal structure. The popular embodiments of this type of sensors can be found under the title “flueric sensors” in [4].

Vortex rate gyroscopes use a concept near to that of jet flow gyroscopes. However, a vortex rate sensor contains a container in which a 2D sink flow is generated. The generated sink flow comprises a vortex whose pattern and streamlines are well-defined, by design, in the absence of an input angular rate. However, when an angular rate is applied to the sensor an additional vortex flow is superimposed to the initial sink flow, which leads to a combined vortex flow. The variation in the fluid streamlines indicates the magnitude and direction of the input rate. There are different designs that can achieve an operating sensor utilizing the vortex rate concept (see [11,12,13,14,15,16,17,18,19,20]).

Integrating angular accelerometers, sometimes referred to as fluid rotor angular rate sensors, are fluid-based angular rate sensors which operate as per the law of conservation of angular momentum (i.e., Navier–Stokes equations), and the continuity equations for viscous laminar Newtonian fluid flows. The typical internal structure of these kinds of sensors includes an annular channel where fluid flows. The fluid flows under the impact of external angular motion. The annular channel is built such that it contains an obstruction or float. The obstruction/float are moved under the impact of fluid flow. The motion of the obstruction/float is an indication of external angular acceleration imparted to the sensor. The motion of such obstruction/float is sensed using bridge circuits or other equivalent pick-off mechanisms. However, the angular acceleration values are integrated by deploying a signal conditioning mechanism to measure an integrated angular acceleration sensor. In other words, the sensor measures the applied angular rate. There were numerous attempts that used the same concept with different embodiments and modifications to achieve angular rate sensors. Among these attempts are a series of US patents that were filed mainly in the 1960s and 1970s [21,22,23,24,25].

A micromachined biomimetic fluid rotor angular rate sensor was introduced by Andreou et al. in 2014 to be used for vestibular prostheses [26]; whereby, the sensor is used as a prostheses equipment that is utilized for people with a malfunctioning vestibular system [27]. The vestibular system is the part of the human body that is used to detect the head motion in space which is a crucial function for self-motion and body balance, adjusting body posture, and stabilizing vision during movement.

There are other types of micro-machined fluid inertial sensors that were introduced twenty years ago. In comparison to MEMS-based IMUs, fluid-based inertial sensors use fluid instead of a solid proof mass to detect the inertial forces. Consequently, the fluid-based inertial sensors are advantageous in terms of having a simple structure, low cost, high shock resistance, and large measurement ranges; whereas, the sensitivity and the bandwidth of the fluid-based inertial sensors are less competitive with the MEMS-based inertial sensors [28].

These micro-machined fluid gyroscopes are based on either jet flow or thermal flow. However, micro-machined fluid gyroscopes are less mature. The jet flow gyroscopes use a laminar gas flow driven by a micro pump, whereby an external rotational motion deviates the flow with an amount proportional to the angular rate by which the rotation was applied [28]. The micromachined jet flow gyroscopes follow the same operation concepts as the previously mentioned jet flow gyroscopes, however, with micromachined structures. Additionally, thermal gas gyroscopes use a flow that is induced by thermal convection. The thermal gas gyroscopes apply the same concept used for thermal accelerometers, where the thermal sensors are placed in a 2D configuration to measure rotation rates instead of linear accelerations [28].

Fluid-based angular accelerometers share most of their technologies and operation concepts with the fluid-based angular rate sensors. However, in their design, fluid-based angular accelerometers lack the signal conditioning mechanism that would integrate their output signal to be an angular rate signal. Nonetheless, fluid-based angular accelerometers can be thought of as the predecessors of fluid-based angular rate sensors; whereby, fluid-based angular accelerometers are usually modified to reach fluid-based angular rate sensors. Consequently, the classification of fluid-based angular accelerometers technologies shares most of its nomenclature with the earlier mentioned angular rate sensors technologies.

One of the first fluid-based angular accelerometer technologies was the fluid rotor angular accelerometer. Fluid rotor angular accelerometers are classified in literature into liquid rotor angular accelerometers and gas rotor angular accelerometers. Furthermore, the liquid rotor accelerometers can be further classified in terms of their motion pick-off mechanism. Liquid rotor angular accelerometers with an electric pick-off mechanism were first introduced in 1957 by Statham laboratories [29]. This was then followed by a modified design of a liquid rotor angular accelerometer with an electric pick-off mechanism reported by Morris et al. in 1970 [30]; whereas, liquid rotor angular accelerometers with a pressure sensing pick-off mechanism was introduced by Amlie in 1964 [31] and Schiltz in 1971 [32]. Gas rotor angular accelerometers operated on a similar concept for that of liquid rotor angular accelerometers. However, gas rotor angular accelerometers mainly utilized a pressure sensing pick-off mechanism, which was introduced by Thomson et al. in 1966 [33] and MacDonald in 1974 [34].

With the revolution of micromachining industries, micromachined and miniaturized versions of the liquid rotor angular accelerometers emerged, such as the one reported by Wolfaardt and Heyns in 2008 [35]. Another version of these micromachined angular accelerometers was reported by Arms in 2003 [36].

Another scientific concept that was used to realize fluid-based angular accelerometers was the heat-mass transfer of fluids. The concept was also utilized for manufacturing angular rate sensors, as stated above. Thermal flow angular accelerometers were reported initially by Benedetto and Linder in 1980 [37,38].

Recently, micromachined biomimetic versions of the thermal flow angular accelerometers were reported by a research group at the University of Twente in 2014, to use these sensors to provide low cost inertial sensors. The reported thermal flow used water as its fluid mass and used the thermal variations of the fluid flowing in an annular channel to derive the angular acceleration imparted to the sensor through solving Navier–Stokes equations. Nonetheless, the reported sensor provided poor performance which cannot be used for vestibular protheses [39].

The same research group at the University of Twente reported a micromachined biomimetic MHD flow angular accelerometer, which was also intended to be a low-cost sensor that utilized tap water and saltwater solutions as fluid mass flowing in microfluidic annular flow channels. The flow was induced when the sensor was subjected to angular motion, and the flow was detected and measured utilizing the MHD effect. However, the sensor also reported a poor performance. 

## 3. Problem Statement

The rate-integrating gyroscopes include various typical error processes in their output. Such error processes include g-insensitive biases, g-sensitive biases, isoelastic biases, scale-factor errors, cross-coupling errors, and zero-mean random biases. Each error process has its own sources, which are discussed thoroughly in [4]. Nevertheless, the rate-integrating gyroscopes are characterized mainly by being relatively more sensitive to linear and angular accelerations and platform vibrations. Moreover, the electromagnetic pick-off elements and torque motor are prone to deviations from external magnetic fields. Hence, rate-integrating gyroscopes demand proper magnetic shielding to prevent such errors from occurring. Additionally, the rate-integrating gyroscopes are highly affected by temperature variations which lead to variations in the magnetic properties of the magnetic materials within the sensor. Such variations lead to first, second, and third order scale-factor errors [4].

Magnetohydrodynamic angular rate sensors require proper magnetic shielding for the indication component to ensure proper rate measurement. Otherwise, the sensors suffer from high bias instability due to their dependency on the magnetohydrodynamic effect.

Fluid rotor angular rate sensors suffer from low bandwidth and low dynamic range in comparison to other angular rate sensors. Nonetheless, the literature did not provide proper performance assessment for such sensors, especially for navigation applications.

In the early developments of these kinds of sensors, the major problems were achieving adequate stability, resolution, and insensitivity to environmental impacts especially temperature variations. Consequently, the fluid-based sensors of this form were not considered as plausible sensors to be used for navigation applications.

Micromachined fluid-based gyroscopes suffer from drift due to improper thermal compensation and vulnerability to variations in ambient temperature. Furthermore, micromachined fluid-based sensors suffer from low bandwidth and low sensitivity. Moreover, the main research aspects to be considered for micro-machined inertial sensors are enhancing temperature compensation, developing a full monolithic IMU, and reducing the cross-coupling errors [3,28].

Angular accelerometers are mathematically incompetent relative to angular rate sensors. The reason lies within the mathematical integration process that is required to retrieve the attitude parameters of a moving platform. Such an integration process over time takes place once for angular rate sensors; whereas, the integration process takes place twice for angular accelerometers. Typically, the integration of the sensor output signal over time acts as a low-pass filter, which eliminates short-term errors such as white noise within the sensor measurements. However, the integration cannot handle nor eliminate long-term errors, such as the bias-instability or in-run variations within a sensor output signal. Consequently, it is preferable to implement the integration process as little as possible to evade long-term error accumulation within the derived quantities and numerical integration truncation errors due to digital signal acquisition from modern sensors.

Moreover, what makes angular accelerometers most unsuitable for inertial navigation applications is an inherited error within all angular accelerometers, known as the threshold error [40]. The threshold is the least angular acceleration value that the sensor can detect under normal operating conditions. The threshold error is defined as the drift that occurs in the computed attitude angle, which occurs when the angular accelerometer is subjected to an angular acceleration value beneath the sensor threshold value. Consequently, an angular accelerometer subjected to an angular rotational motion, about its sensitive axis, with an angular acceleration value lower than the threshold, would result in a drift angle that takes a quadratic form as a function of time, due to the integration process. Similarly, the angular rate sensors might suffer from the same error; however, the resulting drift angle would drift linearly with time [6]. 

As established earlier, angular accelerometers are of low performance in comparison to angular rate sensors, especially for navigation applications; however, some fluid-based technologies that are utilized in the manufacture of angular rate accelerometers are prone to errors that degrade the performance of such sensors even more. The main drawback of liquid rotor angular accelerometers is non-linear responses due to changes in temperature and thermal gradients across the sensor. The changes in ambient temperature can be compensated using volume compensators. However, the sensors should be carefully designed and provided with proper thermal shielding to avoid such effects. Moreover, some designs are sensitive to linear accelerations, which induce errors in the measured angular accelerations. Similarly, the gas rotor angular accelerometers suffer from temperatures gradients across the sensor. Furthermore, proper shielding for the electronic components is required to avoid stray capacitances from affecting output signals.

To sum up, regardless of the technology used to build the inertial sensor, high bias instability is a characteristic of all low-cost inertial measurement units due to either mechanical fabrication imperfections, poor electric pick-off mechanisms, analog to digital signal converters for mechanical sensors, or thermal drift. Additionally, low cost IMUs are characterized by a low signal-to-noise ratio. On the other hand, inertial sensors of high performance are typically of high cost and not free from bias instability in their output.

## 4. Objectives

One objective of the proposed particle imaging velocimetry gyroscope (PIVG) is to eliminate bias instability that is encountered in low-cost inertial sensors. To be specific, all inertial sensors suffer from a drift rate that occurs due to various reasons, regardless of the IMU grade or cost. The main objective of the current invention is to provide a nearly drift-free gyroscope, that provides drift rate values less than the listed values for strategic grade IMUs.

An objective of the proposed PIVG is to eliminate errors that typically occur in the analog to digital conversion process that takes place in any IMU, such as quantization errors. Furthermore, the PIVG does not require signal conditioning to derive the measured angular rate from another measured quantity, such as deriving the angular rate from a measured direct current (DC) voltage, as is the case in most inertial sensors.

Moreover, one objective of the proposed PIVG is to provide an output of a relatively high signal-to-noise ratio (SNR), in comparison to IMUs that are suited for navigation and stabilization applications.

Conforming with fluid-based inertial sensors, the system temperature extremely affects the sensors’ performance. Consequently, it is considered an objective of the current invention to provide a gyroscope that does not include moving parts except for a single particle, which does not cause variation in the temperature of the system and maintains constant throughout the operation time of the sensor, provided that the system assembly considers proper shielding from ambient temperature. Additionally, the proposed PIVG ensures a design that is not affected by variations in the magnetic fields within the environment in which the sensor is operated.

As stated earlier, the cost of an IMU is dependent on the technology and the performance of said IMU, such that the cost increases as the IMU performance becomes better. Hence, the proposed PIVG is of a relatively low cost in comparison to typical IMUs that share the same performance and characteristics.

Additionally, another objective of the proposed PIVG is to be compact for use in typical navigation applications. The design ensures that the PIVG can be mounted for typical navigation platforms, especially in terms of size; such platforms include ground-based, aerial, and marine platforms. Moreover, the design ensures a simple structure that does not require special precautions or procedures in sensor fabrication and operation processes.

## 5. Gyroscope Design

### 5.1. Operation Concept

The proposed fluid-based gyroscope PIVG depends on fluid dynamics to provide the inertial measurements. The concept is that when an inertial force is imposed upon a control volume of fluid, a flow is generated in response to such force to satisfy Newton’s second law of motion. Hence, once the flow is detected, and through the implementation of fluid dynamics theories, the actual inertial measurement can be acquired. The fluid dynamics theories, which are of interest to design this system, are the law of conservation of momentum implemented for incompressible fluids, alternatively referred to as Navier–Stokes equations, and the continuity equation for incompressible fluids. The concept is applicable for either linear or rotational types of motion. However, the PIVG considers the detection and measurement of rotational motions.

From the proposed nomenclature, PIVG employs a version of particle imaging velocimetry to perform the inertial measurements. Particle imaging velocimetry is a branch of fluid dynamics science in which the properties of fluids and fluid flows are determined through tracking particles that are neutrally buoyant and mimic the actual dynamics of the flow. Particle imaging velocimetry implies the use of an imaging sensor that is used to track the particles, whether those imaging sensors are set up in fixed or moving positions along the examined flow. Research has been extensive in particle imaging velocimetry with vast technical advancements in digital image processing to acquire more precise, computationally efficient, and high rates of data. 

However, in this adoption of the concept within PIVG, only one particle is monitored within a predefined control volume flow channel, and the particle monitoring is done using a fixed imaging sensor with respect to the pre-located and fixed flow channel. The motion of such a particle is determined from the acquired sequence of images via a series of digital image processing techniques.

To lay down a basis for particle tracking, the projectivity condition is implemented to determine the relative position of the particle with respect to the image space coordinate system. It is noted that the image space represents a constant view of the flow channel in which the particle is only in motion as result of the fluid flow. Since the object space geometry is known, the use of a single imaging sensor can determine the object space position of any point that is an element of the image space view. Consequently, the position of the particle can be determined directly.

### 5.2. Sensor Design

The adopted design of the PIVG is an open-loop design, in which the sensor performs the measurement without the need of an external restoring mechanism that restores the sensing element to its original state. Nonetheless, the sensing element within the PIVG is designed such that it assumes its original position after each measurement on its own as per the proper damping mechanism provided within the PIVG design.

Any inertial measurement sensor constitutes three main components that are crucial for it to perform the inertial measurement. The components are the motion transduction mechanism, the signal conditioning component, and the sensor readout component. The motion transduction mechanism comprises the sensing element, which is sensitive to the applied external motion to the sensor, and a damping mechanism that eliminates the effects of oscillations and disturbances that occur whenever the external stimulus is unapplied to the sensor. The efficiency of any given damping mechanism is measured by the amount of time required for the sensing element to stabilize after an external disturbing force is removed. The damping mechanism is the mechanism responsible for restoring the sensing element to its stable state (i.e., the state that the sensing element presumes when it is stationary). For closed-loop sensors, the damping mechanism takes the form of external input means that apply an appropriate form of restoring force or couple to the sensing element. However, for open-loop sensors, the damping mechanism relies on the physical properties and the system design to apply the proper damping forces/torques that are required to stabilize the system.

The signal conditioning component is used to apply a modification or adjustment for the signal acquired using the motion transducer and generating a proper output signal for the readout mechanism. The signal conditioning phase might include applying basic operations to the signal, such as filtering, integration, or differentiation. For some sensors, signal conditioning is applied to increase the amplitude or the power of the signal to be able to drive the readout component.

The readout component is a mechanism that transforms the signal into a comprehensive form of information that can be understood by human recognition or can be fed into a controller for mechanical instruments and applications. Hence, any sensor must include a readout mechanism. The readout mechanism for an inertial sensor is usually fed into a controller that can be either used for machine control, platform stabilization, or navigation.

#### 5.2.1. Motion Transduction Mechanism

1. Motion Indicator

PIVG consists of three fluid flow channels placed in three mutually orthogonal axes, with a camera that is facing each flow channel to track a particle that is placed in each flow channel. The particles are chosen to be of the same density of the fluid filling up the flow channels to be neutrally buoyant.

**Figure 1:**
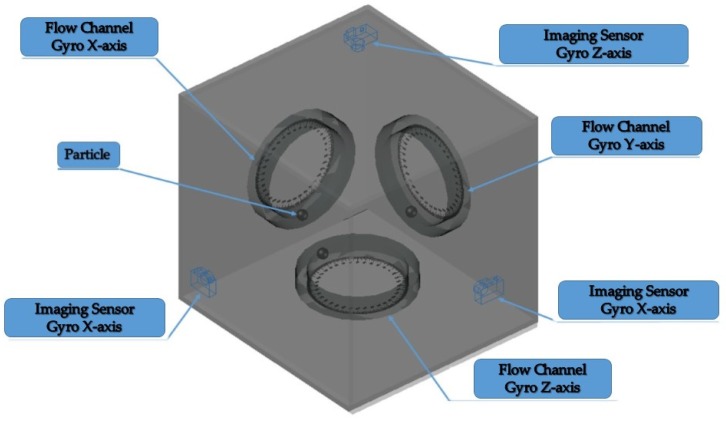
Schematic diagram of a particle imaging velocimetry gyroscope (PIVG) structure.

The described PIVG design represents a three-axis gyroscope; however, the PIVG design can be reduced to be a single axis gyroscope. Figure 1 shows a schematic that shows the system design and structure.

The schematic shows that the PIVG is chosen to be in the form of a cuboid, where there is a flow channel in the shape of a circular torus fixed on each face. There is an imaging sensor facing each flow channel to capture the motion of the particles within each flow channel. For the chosen embodiment of the invention, the imaging sensor is chosen to be a digital camera.

**Figure 2:**
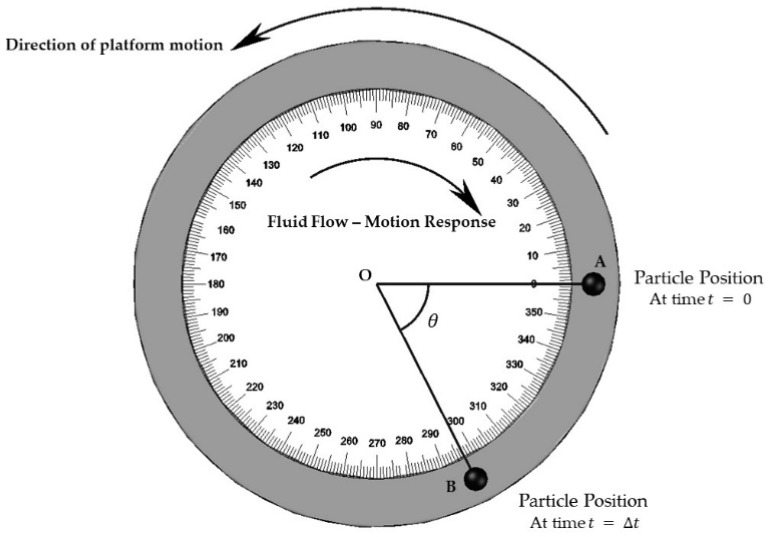
Schematic for flow channel and particle tracking concept.

Once the PIVG is mounted over a moving platform, the imaging sensors capture the motion of the particles through the circular flow channels. The acquired images are then processed to determine the location of the particle. Consequently, the location of the particle within each image is compared with respect to its initial location before motion. To illustrate, the vector ($\vec{OA}$) connecting the initial location of the particle to the center of the circular flow channel is calculated, as shown in Figure 2.

Accordingly, the vector ($\vec{OB}$) connecting the location of the particle at any given instant to the center of the circular flow channel is calculated as well.

The angle confined between those vectors is computed through implementing Equation (1), which is the difference between the slopes of the two vectors ($\vec{OA}$) and ($\vec{OB}$). The computed angle (θ) is proportional to the angular rate of the rotation of the PIVG, and hence, the angular rate of the moving platform.

(1) $$\theta =\tan^{-1}\left(\frac{Y_{B}-Y_{O}}{X_{B}-X_{O}}\right)-\tan^{-1}\left(\frac{Y_{A}-Y_{O}}{X_{A}-X_{O}}\right)$$

where ($X_{O},Y_{O}$) are the coordinates of the center of the circular flow channel, ($X_{A},Y_{A}$) are the coordinates of the initial particle position, and ($X_{B},Y_{B}$) are the coordinates of the particle position.

2. Damping Mechanism

As stated earlier, the PIVG is an open-loop sensor. Hence, the damping force is applied via the natural without the need for any external feedback system to apply the damping effect. The damping effect is applied through the viscous drag force that is created upon the particle, which is a result of the fluid viscosity, and the friction between the fluid layers with the walls of the toroidal pipe, and with the particle itself. Moreover, the proper damping effect (i.e., the damping time) can be adjusted by manipulating the system design components as discussed hereinafter.

Nonetheless, when the PIVG is operated, and as soon as the external applied angular rate is removed, the viscous drag enforces the particle to reach back to its initial stable position (i.e., the particle’s position before the angular rate was applied). However, it should be noted that the design should be adjusted to reach the required damping effect, whereby, the damping effect can be described in terms of the precision of the particle to return to its stable position, and the amount of time that the particles take to restore to a stable position. The proper damping effect implies a high accuracy of position restoration and the least amount of time for the particle to reach stability.

#### 5.2.2. Signal Conditioning

One of the main advantages of the PIVG is that the sensor does not require any signal conditioning, as per the proposed sensor design. The sensor does not require any piece off additional hardware that is responsible for modifying the acquired signal. Rather the signal is directly transmitted to the readout component.

#### 5.2.3. Readout/Pick-Off Mechanism

For the PIVG, the readout mechanism can be any electronic processing unit that acquires the output of the utilized imaging sensor. The electronic processing unit is used to implement the designated image processing algorithms to acquire the position of each particle within its respective flow channel throughout the sensor operation time. The time series of each particle position is directly the output signal that can be used as an indication for the angular rate by applying the proper signal calibration.

#### 5.2.4. Temperature and Pressure Stabilization

PIVG performance depends on the temperature and pressure stability such that the driving and damping forces governed by the fluid depends on the fluid properties remaining constant throughout the entire operation of the gyroscope. Hence, it is crucial to provide means for stabilizing the temperature and the pressure of the fluid control volume; the pressure is kept constant by the design of a well-sealed fluid torus compartment.

### 5.3. Design Parameters

The system design considers a set of design aspects for each component of the system. The design aspects are chosen based on the system components and the fluid dynamics within the chosen control volume for the fluid. These design aspects are interrelated and cannot be separated, rather they should be addressed holistically and are discussed as follows.

#### 5.3.1. Sensing Mechanism—Imaging Sensors

The current embodiment of the sensor implies the use of an imaging sensor to acquire the output signal for the sensor. Hence, the proper design of the PIVG should propose a set of specifications for the imaging sensors that are used to detect the inertial motion of the particles. Such specifications should include the field of view (F.o.V.), focal length, variable or moving lenses, spatial resolution, and temporal resolution.

However, it is clearly stated that any optoelectronic sensor can be used instead of typical imaging sensors to ensure a smaller size and higher data rate for the PIVG.

#### 5.3.2. Flow Channel Design

The flow channel was chosen to be in the form of a circular torus, as depicted earlier by the schematic in Figure 1. The flow channel, in the form of a circular torus, is analogous to the flow of endolymph (i.e., a solution of sodium) within the vestibular system of a human being. The justification for such a shape for the flow channel is so that the PIVG would be insensitive to linear accelerations as they are cancelled out, due to the circular torus design of the flow channel. 

Moreover, the flow channel should be clear and transparent to go with the chosen sensing mechanism (i.e., digital cameras). The design considerations for the flow channel should also include the overall diameter of the torus and its cross-sectional area. The dimensions of the circular torus pipe should follow the limits of Reynold’s number which ensures a laminar flow. Reynold’s number is a unitless quantity that represents in its abstract sense the ratio between the inertial forces with respect to the viscous force acting upon a fluid flow. The formula to compute Reynold’s number ($R_{e}$) is given by Equation (2), and the upper state for laminar flow is achieved at Reynold’s number values less than 200.

(2) $$R_{e}=\frac{\rho vd}{\mu }$$

where *ρ* is the fluid’s density, *v* is the velocity of the flow, *d* is the cross-sectional diagonal dimension, and *μ* is the fluid’s coefficient of viscosity.

Moreover, the flow channel design controls to a certain extent the damping effect that is applied to the particle. Hence, the cross-sectional area and length of the flow channel are of high importance to the amount of momentum that the fluid is going to have, and hence the amount of force imparted to the particle, which determines how far the particle will flow within the channel. On the other hand, such force is balanced with the viscous drag force of the fluid. Furthermore, the fluid channel cross section can be increased or decreased along a certain length of its total length to ensure that the proper damping effect is applied, and that the PIVG is calibrated in terms of the output angular rate signal.

#### 5.3.3. Fluid Specifications

The fluid that should represent the inertial measurement medium for the PIVG should be addressed within the design phase. Hence, the fluid properties, and more specifically, its density and viscosity, are considered crucial for the PIVG design. The reason behind addressing the fluid properties is that the chosen fluid defines a set of defining parameters for the system performance, among which are sensitivity and dynamic range. Moreover, the effect of temperature and pressure upon the fluid properties is another aspect that should be considered.

Nonetheless, the PIVG concept and scientific basis lay down a set of assumptions for the fluid and its flow nature. The fluid is assumed to be Newtonian and viscous, where its flow should always be maintained to be incompressible and laminar.

The chosen fluid has a huge impact on applying the proper damping effect upon the particle, after an external motion is ineffective upon the PIVG. To be specific, the kinematic viscosity of the fluid is crucial to determine the amount of viscous drag that would be imparted to the particle, and subsequently, the damping time and magnitude. Whereby, the kinematic viscosity for a fluid is defined as the ratio between the fluid’s dynamic viscosity at a given temperature, to its density at the same temperature. Hence, it is plausible to vary the used fluid to accomplish a prerequisite sensitivity and damping properties for the PIVG.

#### 5.3.4. Particle Design

It has been established that the material of the particle to be tracked should be of the same density of the fluid to ensure its neutral buoyancy. Nonetheless, the shape of said particle, as well as its dimensions, should be defined with respect to the chosen design for the flow channel.

For the preferred embodiment of the PIVG, the particle is chosen to be spherical, such that it provides a nearly neutral effect upon the driving and the damping forces. Thus, the system response is left to be controlled through the fluid properties and chosen geometry for the control volume (i.e., torus).

The dimensions should ensure that the particle lies at the point of maximum velocity within the velocity gradient of the cross section of the flow channel and at the same time does not block the fluid flow.

Furthermore, the dimensions (i.e., the size) of the particle should be chosen such that it is the least likely to be represented and detected within an image as per the spatial resolution of the deployed imaging sensors. It can be stated that the precision of the detection of the centroid of the particle within any given image would affect the PIVG measurement precision. Hence, as the size increases, the uncertainty in the detection of the centroid of the particle increases accordingly. Therefore, the choice of an appropriate size for the particle should balance between being apparent in the image space and being as small as possible to minimize the uncertainty in its detection.

Moreover, the shape and size of the particle determines, in association with the fluid flow channel design and the type of fluid, the amount of damping force that can be applied to the particle, and hence, to the system overall. The size (i.e., the dimensions) of the particle should be chosen such that the ratio between the cross-sectional area of the particle to the cross-sectional area of the fluid flow channel is optimized to obtain the designated damping effect. Additionally, it is obvious that the shape of the particle is also a basic factor in defining the viscous drag force created upon itself, when interacting with the fluid layers, whereby, the viscous drag force is factored by the impact of the shape around which the fluid is flowing. Nonetheless, the shape of the particle is studied, as well, to optimize the damping effect within the PIVG, as per the design implications.

#### 5.3.5. Digital Image Processing

Digital image processing comprises a crucial aspect in defining PIVG performance. The precision by which the chosen digital image processing algorithm can determine the centroid of the particle defines different PIVG performance parameters including the signal-to-noise ratio (SNR) and the angular random walk.

**Figure 3:**
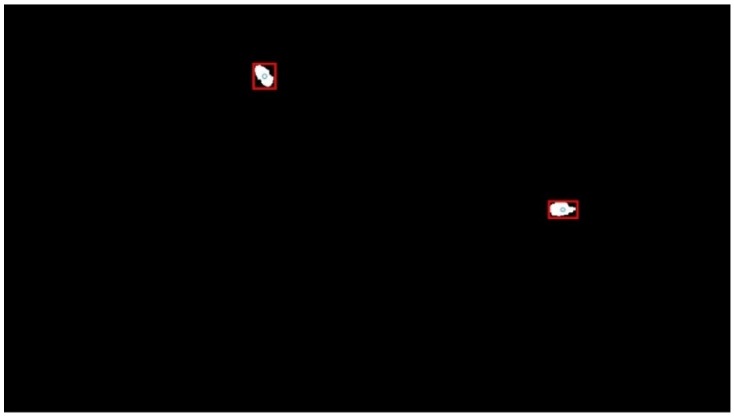
Sample output from digital image processing of the initial phase.

The chosen digital image processing algorithm includes two sequential phases. The initial phase is motion detection of the particle. The initial phase depends on the fact that the image space view is constant and still, except for the particle in the case of external motion. Hence, the initial phase implies computation of the sum of absolute differences of any given image during PIVG operation with respect to the initial image at the beginning. Consequently, the approximate location of the particle, at the initial and current epochs, within the vicinity of the image space can be determined by setting a threshold and binarizing the image as per such threshold. Those locations would appear as white blobs within the black background of the binary image. Figure 3 shows a sample figure of the output of the initial phase. The centroid of each blob is acquired easily by applying binary image labeling.

The second phase is based on the output of the initial phase, such that the computed centroid is used to create a region around itself with the dimensions of the bounding box of such a blob, as depicted by Figure 3. Such a region is used as a sub-image for further processing. Initially, the red, green, blue (RGB) values within that region are compared to a reference RGB value which is predefined based on the color of the used particle. Once the region satisfies such conditions, edge detection is implemented to define the outer edge of the particle. The outer edge is fitted to the actual geometric shape of the particle using least squares adjustment. Finally, a more precise centroid is computed for the particle. The algorithm is repeated sequentially for each acquired image during the PIVG operation time.

As explained earlier, the angle confined between the vector connecting the current particle position and the center of the circular torus projection within the image, and the vector connecting the initial particle position and the center of the circular torus projection within the image space, can be computed.

### 5.4. Sensor Embodiment

The following section shows the current embodiment of the sensor. It is noted that the current embodiment of the sensor is subject to variations and modifications to reach the optimum sensor design, in terms of cost, structure simplicity, size, and performance. The PIVG, as stated earlier, is introduced as a triaxial gyroscopic assembly, which can be adopted to a single axis, as per the application requirements. Nonetheless, the invention embodiment would assume the triaxial configuration for the sake of generalization, with emphasis on the PIVG being adaptable for inertial navigation applications.

**Figure 4:**
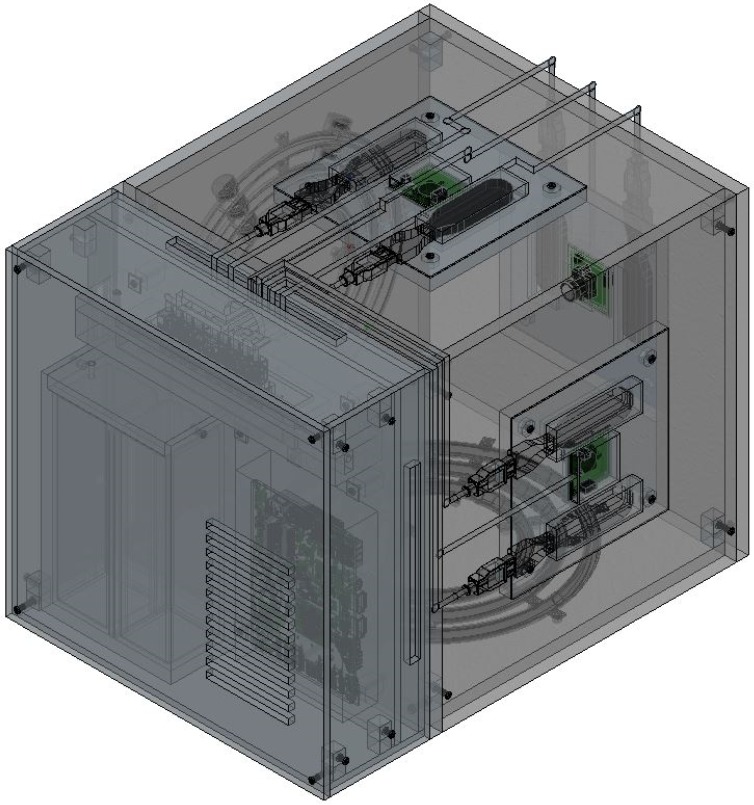
Three-dimensional (3D) perspective view of a PIVG.

**Figure 5:**
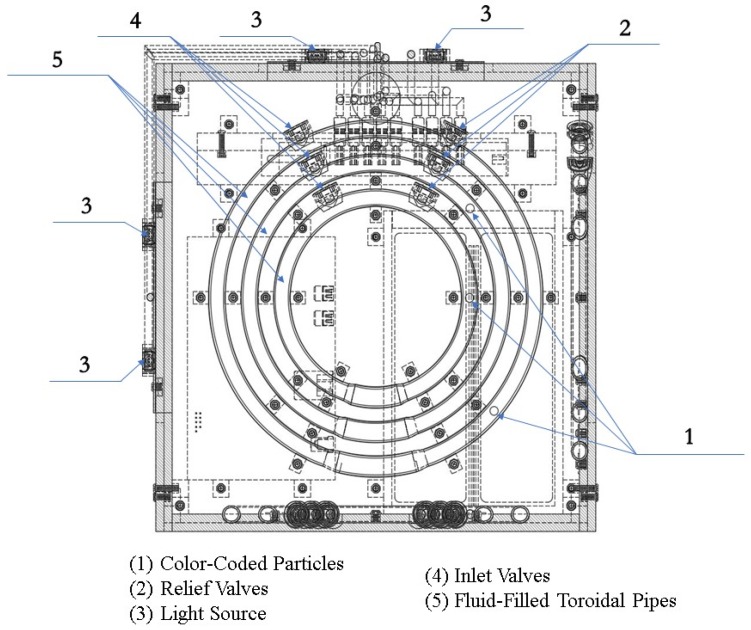
Elevation section of a PIVG.

**Figure 6:**
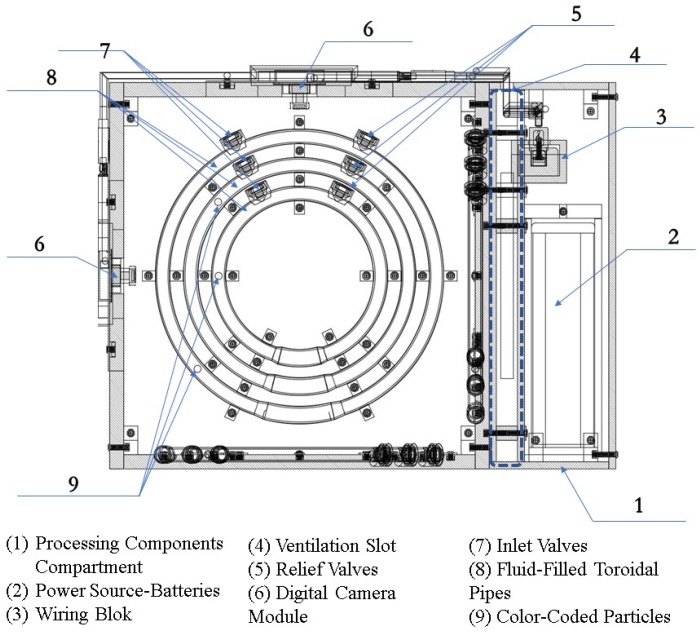
Side view section of a PIVG.

**Figure 7:**
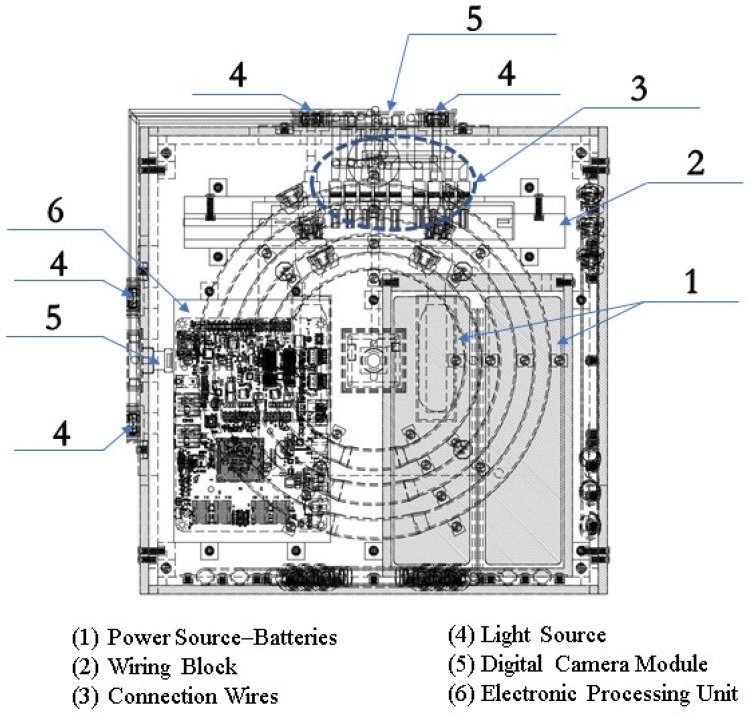
Back section of a PIVG.

Figure 4 shows a 3D perspective view of the PIVG assembly, along with a section view of some parts, to provide insight on the sensor in its final form. Figure 5 shows an elevation section through the PIVG and Figure 6 shows a side section view of the PIVG assembly. Figure 7 shows a back section through the PIVG.

As shown in the above figures, the sensor comprises a set of three perpendicular planes in the form of a cube, whereby each plane constitutes a set of three concentric fluid flow toroidal channels. The fluid flow channels are made of transparent material such as glass, acrylic, or an equivalent material that ensures transparency and sufficient material strength to resist vibrations and shocks. Additionally, the material must allow manufacturing tolerances that ensure the fluid flow channel can be hermetically sealed to preserve the pressure of the contained fluid at a constant value. Each fluid flow channel has a fitted valve that is used for filling the channel with fluid, and as an entry passageway to the particle. As explained earlier, each flow channel is filled with a fluid that is chosen to optimize the performance of the PIVG. A color-coded spherical particle is inserted within each fluid flow channel, such that the particle is neutrally buoyant, and with specific known dimensions. The material of each particle is chosen such that the particle manufacturing process should ensure a smooth surface for the particle, such that it minimizes the chance of creating micro turbulences around the particle, which might affect the particle motion within the channel. The particles are color-coded to facilitate and ensure the potency of the digital image processing phase within the PIVG operation.

The rationale behind adding another two toroidal flow tubes within each measurement plane of the PIVG is to increase the overall sensor sensitivity for angular rate measurements. In such a case, each fluid flow channel contains a different fluid, has different dimensions, and contains particles with different shapes and size, whereby the system of flow channels provides angular rates around its concentric axis, with each representing a different range of sensitivity. The particles are tracked within each fluid flow channel, and the proper particle to be considered is determined by applying the digital image processing algorithm as explained earlier. The processing algorithm defines the time series of the location of each particle within the set of concentric flow channels. For each particle, the motion rate is determined instantly. Afterwards, a particle reflecting an insensitive reaction to a low dynamic motion or a particle showing excessive hyper-reaction to a high dynamic motion is eliminated from the computation process of the angular rate. It should be noted that a particle is defined as reactive to external applied motion based on a prior set of calibration tests performed on the PIVG.

Another attribute of a set of concentric toroidal fluid flow channels for each measurement axis is to provide redundancy in measurements, higher dynamic range, and higher dynamic sensitivity. Moreover, they can act as checks for proper measurements. Hence, the processing algorithm is supplemented with an additional phase by applying a proper estimation algorithm that ensures that the PIVG provides the best estimate of an angular rate.

For each a plane within the PIVG that contains a set of fluid flow channels, there is an opposing plane that contains a high-resolution small-sized digital camera module, with a high frame rate and large F.o.V. Preferable digital camera modules have an F.o.V. within 80° to 120°. Adjacent to the digital camera module, on any given face, a pair of light sources is mounted, one on each side of the digital camera module. The light sources are used to illuminate the fluid flow channels. Consequently, the triaxial PIVG should contain a set of three digital camera modules, each placed on a face opposite a face containing the fluid channels. Additionally, the triaxial PIVG contains a set of six light sources. The assembly of digital camera modules and light sources are connected to a wiring block that facilitates the connection of the electronic elements to the on-board electronic processing unit. The on-board electronic processing unit is used to implement the designed processing algorithm in real time mode.

The electronic processing unit is then connected to an interface, which is considered as a controller used for providing feedback for a machine, or robot, or can be used for performing automated mobile navigation and mapping. The interface also includes a monitor that provides the measurements for instantaneous visualization and a storage module for data storage. The total assembly of electronic modules are connected to a power supply as shown in Figure 7.

The PIVG should be provided with a thermal compensation mechanism that is utilized to account for the change in ambient temperature and internal temperature variations. The thermal compensation mechanism comprises a set of internal ventilation passageways that isolate the fluid-contained compartments, as depicted by Figure 6. The thermal compensation mechanism includes a thermal sensor that measures the system temperature within the PIVG. The measured temperatures are fed back into the processing unit to apply a thermal compensation to the acquired angular rate measurements. The thermal compensation model is predetermined for the PIVG through a series of calibration tests that are performed as an integral part of the PIVG manufacturing process.

## 6. Experimentation and Validation

**Figure 8:**
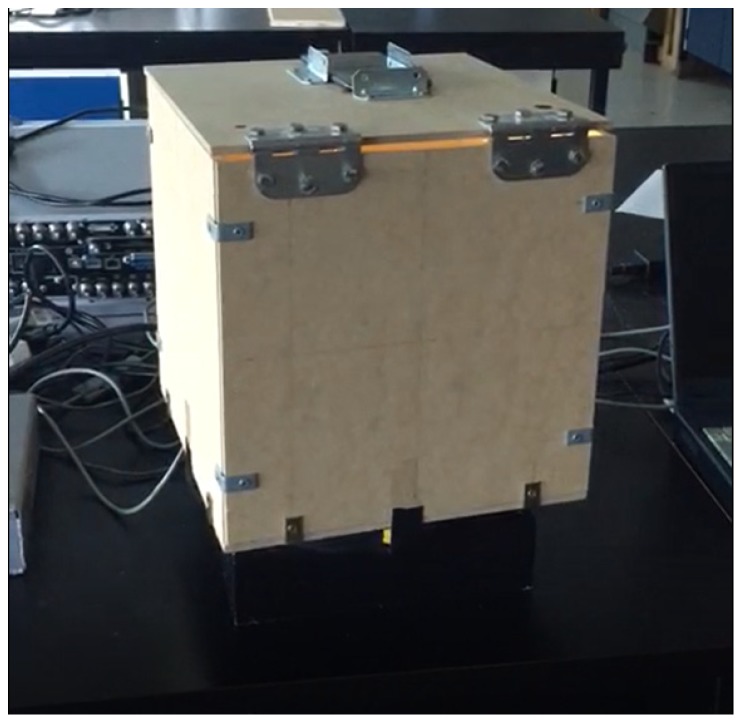
Initial PIVG prototype.

To validate and prove the concept of the PIVG, a series of experiments were held to determine the performance of a prototype of the PIVG. The prototype comprised only a single toroidal fluid-filled channel instead of three channels. Figure 8 shows an image for the PIVG prototype that was used for experimental validation.

The preliminary design of the initial PIVG prototype used for this experiment was a single axis gyroscope design. The body of the adopted single axis PIVG was fabricated as a wooden box in the shape of a cube with a side length 30 cm. The circular torus flow channel is made of a transparent vinyl flexible pipe with an overall diameter 18 cm and a cross-sectional diameter of 1.30 cm. The flow channel is fixed to the bottom of the wooden box. On the opposite face to the flow channel, a smartphone’s camera is mounted to be used as the imaging sensor. The smartphone used in this experiment is LG X-power smartphone, and Table 1 shows the specifications of the digital camera mounted on the smartphone. It is noted that the camera was used in video mode throughout the entire set of tests.

The used fluid is a water sodium-hydrochloride (NaCl) solution with 0.7% concentration. The solution was realized with a mixture of ordinary tap water and table salt. The reason behind using a NaCl solution is to achieve a fluid density that is equal to the density of the particle which was used. The particle used was a plastic hollow sphere of with a diameter of 5 mm and relative density of 1.1 gm/cm^3^.

The experiments mainly address the sensor response validity in terms of amplitude and SNR. Furthermore, the experiments address the hypothesis that the PIVG is nearly drift-free, and that the sensor can maintain substantially long periods of time with no induced errors in the PIVG output signal.

### 6.1. PIVG Response Validation

The objective of this experiment was to test the PIVG response to external angular rates. The experiments were designed to address the amplitude response of the PIVG for a given dynamic range. The amplitude response of a sensor is a measure of system interaction under the impact of the external effect. It is a measure of a system’s ability to faithfully sense and transmit the information required from the sensor to measure.

#### 6.1.1. Experimental Setup Description

The experiments were held in a laboratory where the PIVG was placed and centered on a servo driven turntable. The used turntable is a high-end turntable that is used for precise calibration and angular rate tests for angular rate sensors. The turntable has two degrees of freedom (D.o.F.) and is controllable via a PC-connected controller. It can be operated within a wide range of input angular rates, up to 220°/s, and a range of operating angular accelerations, up to 50°/s^2^. Nonetheless, for these experiments, the turntable was used a single degree of freedom (D.o.F.) setup to test a single axis of the PIVG.

To be more conservative, and to validate the PIVG measurements, the experiments were performed simultaneously with another reference gyroscope mounted on the turntable. The reference gyroscope is Xsens MTi-G-710 GNSS-aided IMU, which is a stable commercialized sensor that is used for inertial navigation.

The turntable was used to test a single axis of the PIVG over a series of experiments at different angular rates that were chosen to test the operability of the PIVG for the dynamic range encountered by most typical navigation and mobile mapping platforms. Moreover, the PIVG was tested for its response for different values of angular accelerations to address the sensitivity of the PIVG angular rate measurements to angular accelerations. The tests for the effect of angular acceleration are referred to as centrifuge tests [3]. Nonetheless, the input angular rates were within the range 10°/s to 180°/s, while the angular acceleration values ranged from 10°/s^2^ to 50°/s^2^. The experiments were held such that each value of angular acceleration was fixed while changing the values of the angular rates from 10°/s to 180°/s with increments of 30°/s apart. Consequently, the angular acceleration value was increased by 10°/s^2^ increments and the angle rate tests were repeated. The duration of each experiment was about one minute. Table 2 summarizes the experiments performed on the PIVG in comparison to the reference gyroscope.

#### 6.1.2. Experimental Results

**Figure 9:**
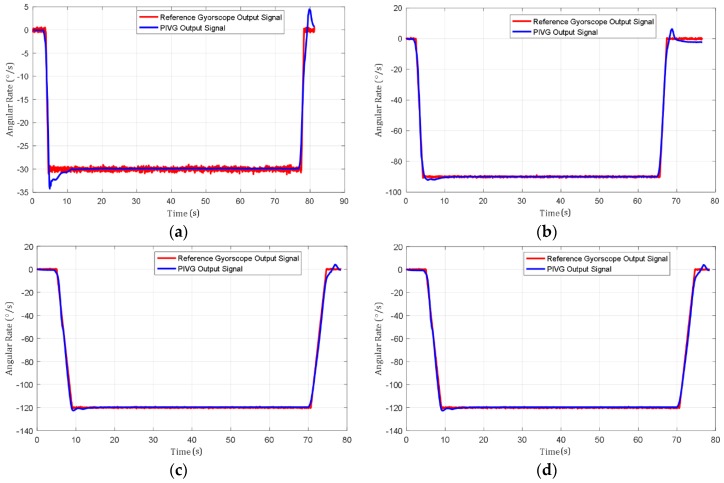
Sample output from the PIVG for different inputs in comparison to a reference gyroscope. (**a**) PIVG output signal versus reference gyroscope at angular rate of 30°/s and an angular acceleration of 40°/s^2^; (**b**) PIVG output signal versus reference gyroscope at an angular rate of 90°/s and an angular acceleration of 50°/s^2^; (**c**) PIVG output signal versus reference gyroscope at an angular rate of 120°/s and an angular acceleration of 30°/s^2^; (**d**) PIVG output signal versus reference gyroscope at an angular rate of 120°/s and an angular acceleration of 20°/s^2^.

Figure 9a–d shows a sample of the experimental results acquired from the series of angular rate tests performed by the PIVG in comparison to the reference gyroscope.

**Figure 10:**
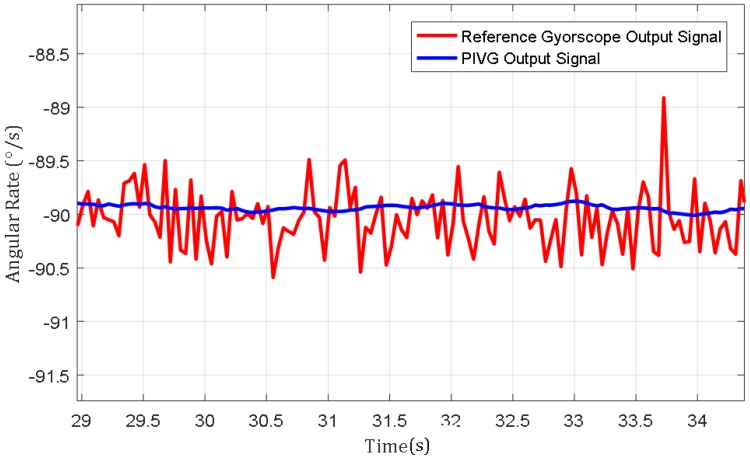
An excerpt of nearly five seconds from the experiment held at an input angular rate of 90°/s and angular acceleration of 50°/s^2^.

Figure 10 shows an excerpt of nearly five seconds from the experiment held at an input angular rate of 90°/s and angular acceleration of 50°/s^2^. The figure emphasizes the difference between the SNR for the PIVG output signal in comparison to the reference gyroscope output signal.

The results show that the PIVG provided substantially accurate results in comparison to the input angular rates from the turntable controller and the reference gyroscope results. Additionally, the PIVG processing model demonstrates substantially a proper response with minimal delay in comparison to the reference gyroscope response and shows that the PIVG is not affected by variations in angular accelerations. Moreover, the results show that the PIVG has a relatively high SNR in comparison to the reference gyroscope.

Finally, the results of the experiments have shown the repeatability of the PIVG results under different operating conditions. Hence, the PIVG can be used as a reliable angular rate sensor for various applications, as discussed earlier. However, the PIVG must pass through a series of calibration tests that are crucial to ensure the performance that is shown by the above results. The calibration tests account for the sensor modeling parameters and environmental operation conditions.

### 6.2. PIVG Drift-Free Validation

The main objective of this invention was to have a nearly drift-free angular rate sensor, as stated earlier. The objective of this experiment was to validate that the PIVG is a nearly drift-free angular rate sensor, which can operate for substantially long periods of time without the occurrence of drift rate within the output angular rate signal. Typically, there is no angular rate sensor that does not have a component of drift rate within its output; however, such drift rate is minimal in high-end inertial sensors. On the other hand, these sensors tend to be of an extremely high cost. The PIVG is validated through this experiment to be the first nearly drift-free inertial sensor at an extremely low cost, in comparison to sensors of comparable performance, especially in terms of drift and signal stability.

#### 6.2.1. Experimental Setup Description

The experimental setup of this experiment is like the experimental setup discussed for the previous set of validation experiments. However, the PIVG was mounted in the turntable for a single axis experiment. The duration of the experiment was nearly five hours of operation, where the turntable was rotated at an angular rate of 60°/s. The data rate of the PIVG was set to 24 Hz through the frame rate of the utilized camera module. Hence, the total number of samples acquired by the PIVG was 472,769 samples.

#### 6.2.2. Experimental Results

**Figure 11:**
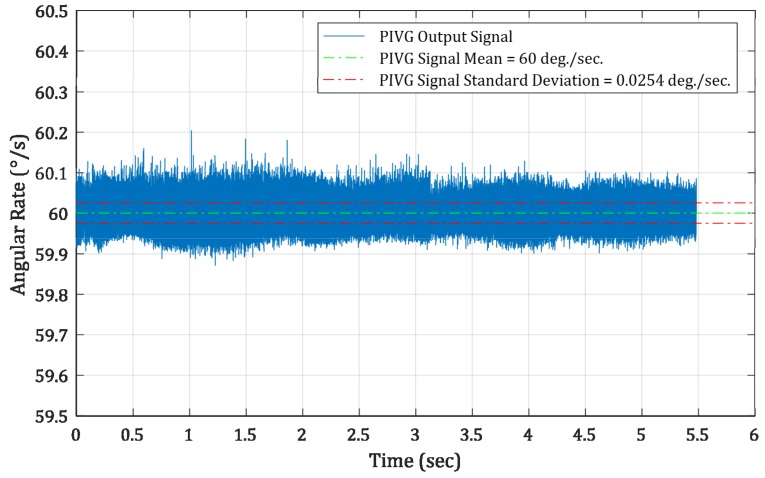
The result of the drift-free validation experiment.

Figure 11 shows the result of the drift-free validation experiment. The figure shows the stable portion of the output signal at 60°/s, after excluding the start-up portion in which the turntable had to reach its maximum rate from rest to illustrate the drift-free signal acquired by the PIVG.

From the signal shown in Figure 11, the error signal can be computed by subtracting the mean of the output angular rate values from the signal. The result shows a zero-mean error signal with a standard deviation of 0.0254°/s.

Consequently, it is obvious from these results that the PIVG has no drift rate in its output signal over an operation period of nearly five hours, which covers most of the requirements of inertial navigation applications at a relatively low cost. It is also noted that the SNR (shown in the results of this experiment), although comparable to the current state of the art angular rate sensors, can be enhanced by using better imaging sensors with higher spatial resolution.

## 7. Discussion

To conclude, this paper introduces a fluid-based angular rate sensor that is nearly drift-free with a high SNR. Such a fluid-based inertial sensor is realized at a relatively low cost in comparison to systems of the same performance. The introduced fluid-based angular rate sensor is referred to as PIVG. PIVG utilizes fluid as the inertial mass and operates on PIV concepts.

A prototype for the PIVG was designed and fabricated as a proof of concept. The prototype was tested against a commercial industrial grade IMU. The results showed the repeatability and accuracy for rate transfer tests. In addition, the results also showed that the PIVG is nearly drift-free; whereas, the PIVG recorded a zero-mean error signal with a standard deviation of 0.0254°/s for an operation period of nearly five hours.

Research is ongoing to better model the PIVG response and calibrate the various systematic errors and model any stochastic errors that are encountered with the use of the PIVG.

## 8. Patents

This research entails a pending patent; however, research is currently ongoing to achieve a fully functional prototype for the PIVG [41].

## Figures and Tables

**Table 1:** Smartphone LG X-power digital camera specifications.

Specification	Value
Field of view (along short dimension)	33.4°
Focal length	4.92 mm
Frame rate	30 fps
Lens group	Moveable—auto focus enabled
Resolution	1280 × 720

**Table 2:** Response validation experiments on a PIVG.

		Input Angular Accelerations (°/s^2^)				
		10	20	30	40	50
**Input Angular Rates (°/s^2^)**	10	(✓)	(✓)	(✓)	(✓)	(✓)
	30	(✓)	(✓)	(✓)	(✓)	(✓)
	60	(✓)	(✓)	(✓)	(✓)	(✓)
	90	(✓)	(✓)	(✓)	(✓)	(✓)
	120	(✓)	(✓)	(✓)	(✓)	(✓)
	150	(✓)	(✓)	(✓)	(✓)	(✓)
	180	(✓)	(✓)	(✓)	(✓)	(✓)
